# Limb regeneration in a direct‐developing terrestrial salamander, *Bolitoglossa ramosi* (Caudata: Plethodontidae): Limb regeneration in plethodontid salamanders

**DOI:** 10.1002/reg2.93

**Published:** 2017-12-06

**Authors:** Claudia Marcela Arenas Gómez, Andrea Gómez Molina, Juliana D. Zapata, Jean Paul Delgado

**Affiliations:** ^1^ Sede de Investigación Universitaria Torre 2, Laboratorio 432, Calle 62 No. 52–59 Medellín Colombia; ^2^ Grupo de Investigación en Patobiología Quirón Universidad de Antioquia Ciudadela Robledo, Carrera 75 # 65–87, bloque 47, oficina 134 Medellín Colombia; ^3^ Grupo de Genética, Regeneración y Cáncer Universidad de Antioquia Medellin Colombia

**Keywords:** axolotl, genome size, newt, plethodontid, urodele

## Abstract

Appendage regeneration is one of the most compelling phenomena in regenerative biology and is extensively studied in axolotls and newts. However, the regenerative capacity in other families of salamanders remains poorly described. Here we characterize the limb regeneration process in *Bolitoglossa ramosi*, a direct‐developing terrestrial salamander of the plethodontid family. We (1) describe the major morphological features at different stages of limb regeneration, (2) show that appendage regeneration in a terrestrial salamander varies from other amphibians and (3) show that limb regeneration in this species is considerably slower than in axolotls and newts (95 days post‐amputation for complete regeneration) despite having a significantly smaller genome size than axolotls or newts.

## 1 INTRODUCTION

Tissue regeneration is widely observed among metazoans early in development. Urodele amphibians are unique among vertebrates because they retain remarkable regenerative abilities as adults. Adult urodele amphibians can regenerate highly differentiated tissues and organs such as the heart (Oberpriller & Oberpriller, 1974), jaws (Ghosh, Thorogood, & Ferretti, 1996), intestine (O'Steen, 1958), lens (Reyer, 1956), and complex structures such as limbs (Spallanzani, 1768), which is the most surprising because the limb must regenerate a new functionally integrated structure (McCusker & Gardiner, 2014). During the regenerative process, key events have been described, including the formation of a regenerative epithelium and blastema (Iten & Bryant, 1973; Tank, Carlson, & Connelly, 1976). The regenerative epithelium is a source of secretion of important growth factors that promote the formation and proliferation of blastema cells (Campbell, 2011), which are dedifferentiated cells that proliferate prior to differentiation and rebuilding of the new limb blastema (Iten & Bryant, 1973; Tank et al., 1976).

Axolotls (Ambystomatidae) and newts (Salamandridae) are the most commonly used model organisms (Maden, 2008; Voss, Epperlein, & Tanaka, 2009). Recently, it has been shown that these species have different mechanisms to rebuild tissues during limb regeneration; for example, muscle regeneration in axolotls is led by satellite cells, whereas newt muscles go through a process of dedifferentiation to generate proliferating cells (Sandoval‐Guzmán et al., 2014). The capacity for lens regeneration also changes: whereas axolotls can regenerate a lens only during early developmental stages, newts sustain this ability throughout adulthood (Eguchi et al., 2011).

The regenerative capacity of tissues and appendages in other families of salamanders has not been widely described. The South American salamanders are represented only by the Plethodontidae family, mainly by the genus *Bolitoglossa* (130 species), which is widespread over the American tropics, mainly from northeastern Mexico to the South American rainforest (Parra‐Olea, García‐París, & Wake, 2004). In Colombia, 23 species of plethodontids are reported, 21 included in the *Bolitoglossa* genus (16 endemic species) and two species in the *Oedipina* genus. These species are present in primary and secondary forests of low mountains (from 1200 to 3000 m above sea level) and are arboreal and nocturnal terrestrial animals (AmphibiaWeb, 2017).

Plethodontidae species show some biological differences from the other families such as enucleated red blood cells, a projectile tongue, the absence of lungs (Wake, 2009), tail autotomy (Mueller, Macey, Jaekel, Wake, & Boore, 2004), nasolabial grooves and postaxial development of the digits (Wake & Hanken, 1996). They also undergo direct development, where the embryos develop to an adult without any larval stages (Chippindale, Bonett, Baldwin, & Wiens, 2004).

Limb regeneration in North American species that belong to the family Plethodontidae, such as *Plethodon cinereus*, *P. dorsalis*, *Desmognathus ochrophaeus*, *D. fuscus*, and *Eurycea bislineata*, has been described (Scadding, 1981). In the present study, we describe the complete stages of forelimb regeneration in *Bolitoglossa ramosi* (Caudata: Plethodontidae), an adult terrestrial salamander with direct development.

## 2 RESULTS

### 2.1 Stages of limb regeneration

**Figure 1:**
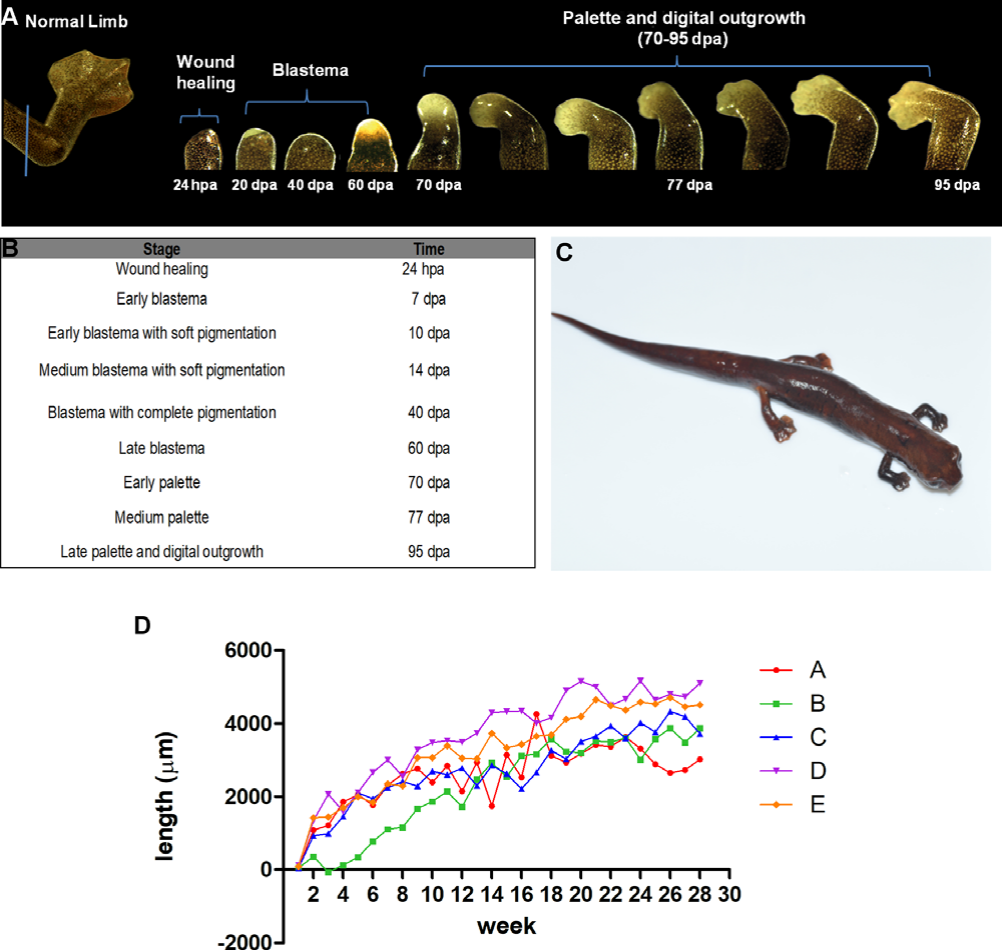
Stages of limb regeneration in *Bolitoglossa ramosi*. (A) The normal limb of *B. ramosi* is webbed and consists of four digits with an average length of 7.6 ± 1.5 mm (blue line indicates mid‐humerus level plane of amputation). Regenerative stages are indicated: wound healing (24 hpa), blastema (20 dpa), and pigmented blastema (40 dpa), where high pigmentation was noticed, and late blastema (60 dpa). The stages of palette and digital outgrowth occur between 70 and 95 dpa. (B) Nine macroscopic stages were clearly observed, showing that digit extension started after 95 dpa. (C) *B. ramosi* after 28 weeks post‐amputation (limb with a length of 5.63 ± 1.22 mm). (D) Limb growth rate per day (e.g., [growth in week 2 − growth in week 1]/7 days) in *B. ramosi* during the 28‐week period, showing the rate for five animals (A, B, C, D, E). hpa, hours post‐amputation; dpa, days post‐amputation

The stages of forelimb regeneration in *B. ramosi* were identified by digitally recording the growth of the regenerate at weekly intervals. We have identified nine macroscopically defined stages (Fig. 1A, B). The normal forelimb of *B. ramosi* has an average length of 7.6 ± 1.5 mm, is webbed, and consists of four digits (Fig. 1A).

The growth rate of the regenerate was higher during the first blastema stages, but by 10 weeks post‐amputation the growth rate had declined further with the onset of differentiation of the blastema. This tendency can be observed in Figure 1D for five animals as indicated by each line in the graph (A, B, C, D, E). We excluded two animals because one died and the other underwent tail autotomy during limb regeneration.

Upon amputation, the animals bled slightly and a clot was immediately formed. Wound healing occurred by 24 h post‐amputation (hpa), which consisted of a thin unpigmented wound epidermis and accumulation of a blood clot under the wound epithelium. A second stage corresponded to an early stage blastema by 7 days post‐amputation (dpa), which was similar to the description of the blastema reported in other salamanders (Iten & Bryant, 1973; Tank et al., 1976). The apex of the regenerate was thickened without any sign of blood clots or inflammation. At 10 dpa, an early pigmented blastema was visible at the distal tip of the regenerate. By 14 dpa, a medium pigmented blastema was observed, and at 40 dpa the pigmentation was increased. At 60 dpa (late blastema), the pigmentation was noticeably lower in the regenerate (Fig. 1A), and blastema growth increased and showed signs of vascularization. By day 70, dorso‐ventral flattening occurred and anterior−posterior axes were evident, showing a palette and elbow formation. At 77 dpa, a palette stage similar to other salamanders was evident with early demarcation of interdigital grooves. A clear extension of the digits was observed 95 dpa (Fig. 1A). After week 28, the regenerative limb measured 5.63 ± 1.22 mm (Fig. 1C). Later stages of limb regeneration corresponded to growth and extension of the digits.

In addition, we compared the macroscopic observations during limb regeneration of *B. ramosi* with *Bolitoglossa vallecula*, another endemic species of the Colombian Andes which shows a similar trend during regeneration and growth (Fig. S1).

### 2.2 Histological observations

#### 2.2.1 Normal limb

**Figure 2:**
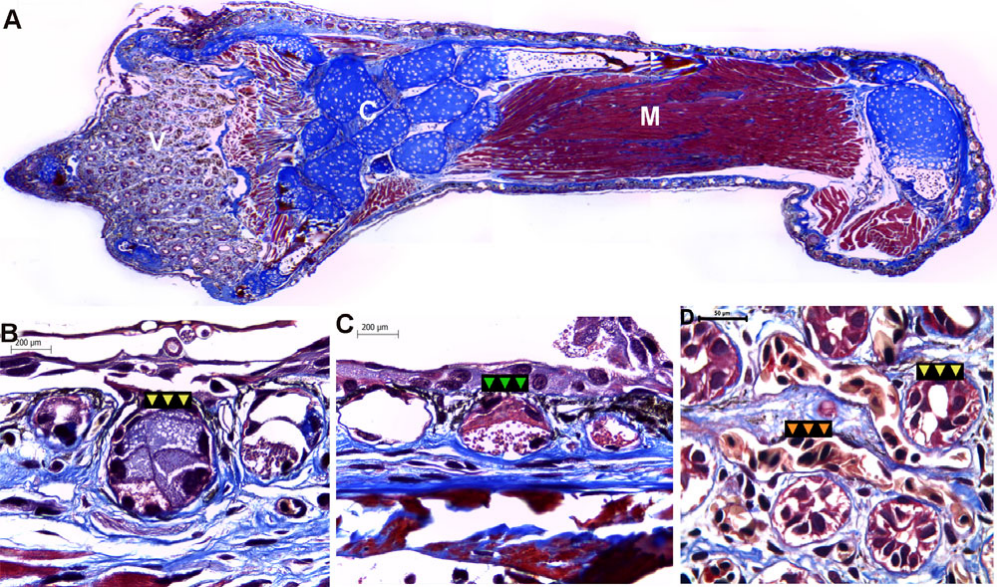
Histological analysis of the adult forelimb in *Bolitoglossa ramosi*. (A) Staining with Masson's trichrome identifies muscles, connective tissues, and condensation of the cartilage. Additionally, the skin of the distal part of the limb is glandular with high vascularization (V). (B)−(C) Subepithelial glands are abundant surrounding the skin, in particular acinar (yellow arrows) and alveolar glands (green arrows) both with mixed secretions (serous and mucous). (D) The skin at the distal part of the normal limb is highly vascularized (orange arrows) and glandular (yellow arrows). V, vascular tissue; C, cartilage; M, muscle

Masson's trichrome staining showed an epidermal tissue composed of two to three layers of epidermal cells. The distal part of the limb was highly vascularized and glandular (Fig. 2A, D); additionally, the presence of subepithelial glands (acinar and alveolar glands) with mixed secretions (serous and mucous secretion) was observed (Fig. 2B, C). The dermo‐epidermal junction showed melanophores (melanocytes) and high pigmentation (eumelanin granules), whereas the superficial dermis was composed of densely packed collagen fibers. The skeletal striated muscle displayed a peripheral nucleus and transverse striations, and the presence of some multinucleated myocytes was observed (Fig. 2A). Additionally, the limb sections showed the presence of cartilage with some ossification in the peripheral regions (Fig. 2A).

#### 2.2.2 Wound healing (3 dpa)

**Figure 3:**
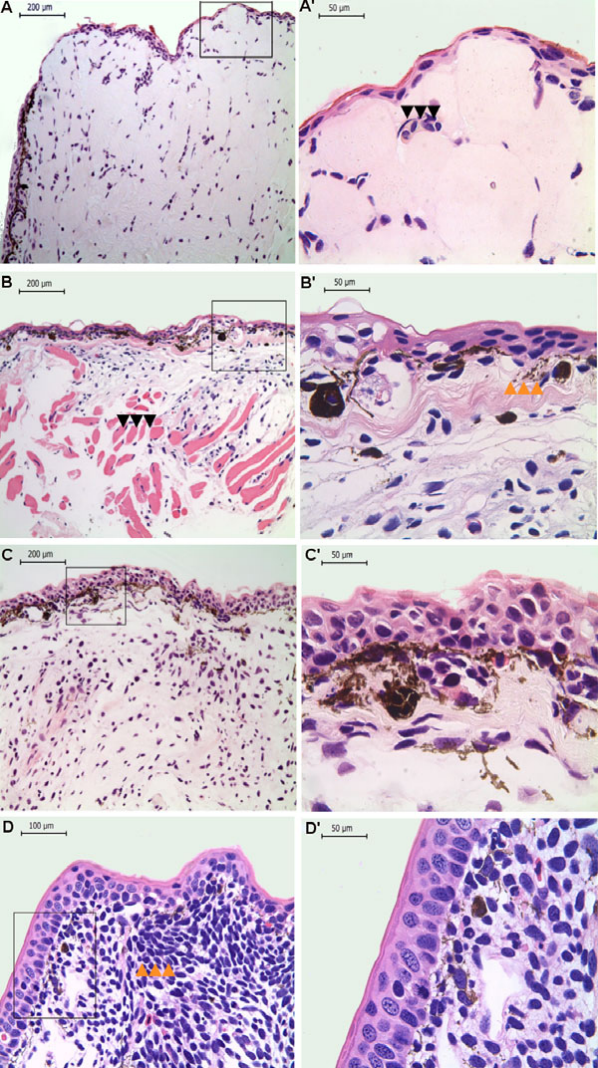
Histological analysis during limb regeneration in *Bolitoglossa ramosi*. Hematoxylin and eosin staining at low magnification (A, B, C, D) and high magnification (40×) (A′, B′, C′, D′). (A), (A′) Blastema 3 dpa. A thin wound epidermis covers the regenerate, and inflammatory cells are present (black arrows). (B), (B’) Blastema 20 dpa. The dermal−epidermal junction shows the presence of melanophores and eumelanin. The striated muscle appears fragmented within the regenerate (black arrows in B). Additionally, the differentiating dermis contains immature collagen fibers, and a basement membrane is present (orange arrows in B′). (C), (C′) Blastema 40 dpa. High pigmentation is visible within the blastema as well as the differentiating epithelium. (D), (D’) Early palette 70 dpa. The epidermis is well differentiated, and mesenchymal cells show condensation (orange arrows in D) and differentiation to form the digit primordium. dpa, days post‐amputation. The squares represent the areas of the figures with high magnification

At 3 dpa the stump surface was covered by a thin wound epidermis, with two non‐definable layers and lack of a clear basement membrane. The tissues beneath the epithelial layer contain disorganized striated muscle fibers and inflammatory and red blood cells. Tissue proliferation, pigmentation, or vascularization was not evident at this point (Fig. 3A, A′).

#### 2.2.3 Early blastema (20 dpa)

The epidermis was two to three cell layers thick, and keratinocytes were disorganized with no clear organization between the layers, but a partial basal membrane was identifiable underneath the epithelial tissues. The dermal−epidermal junction showed aggregation of undifferentiated cells on an edematous collagen stroma, with immature collagen fibers. In addition, melanophores and eumelanin were observed. The striated muscle appeared fragmented within the regenerate, and no inflammatory cells were evident (Fig. 3B, B′).

#### 2.2.4 Pigmented blastema (40 dpa)

**Figure 4:**
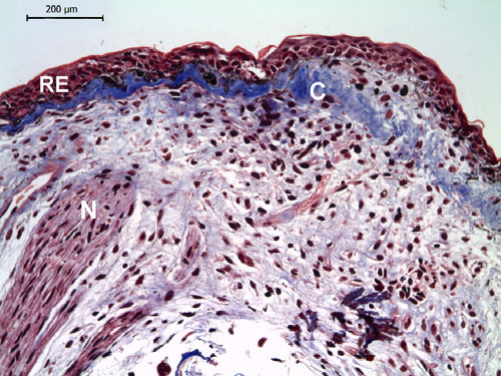
Pigmented blastema at 40 days post‐amputation in *Bolitoglossa ramosi*. The regenerative epithelium (RE) is thickened with multiple layers. The differentiating dermis shows an accumulation of abundant mature collagen fibers (C) and the limb blastema contains undifferentiated proliferating cells. The regenerating nerves (N) extend into the limb blastema. N, nerve; C, collagen deposition; RE, regenerative epithelium; dpa, days post‐amputation

At this stage, the regenerative epithelium was thickened with five to seven layers, and mitotic cells were identifiable within the epidermis. Melanophores and eumelanin were clearly identifiable in the dermal−epidermal junction and the blastema zone (Fig. 3C, C′). The blastema was characterized by the accumulation of immature cells with large nuclei, showing signs of cellular dedifferentiation. A few glandular cells in the superficial dermis were also observed, supported by mature collagen fibers. Additionally, the blastema was innervated by regenerating nerve fibers (Fig. 4).

#### 2.2.5 Early palette (70 dpa)

At this stage, the epidermis was well differentiated with columnar epithelial cells. Melanophores and eumelanin were present throughout the dermal layers. In the regenerate, the mesenchymal cells showed signs of condensation to form the cartilage, and the four‐digit primordium was observed (Fig. 3D, D′ and S2).

### 2.3 Cell proliferation assay

**Figure 5:**
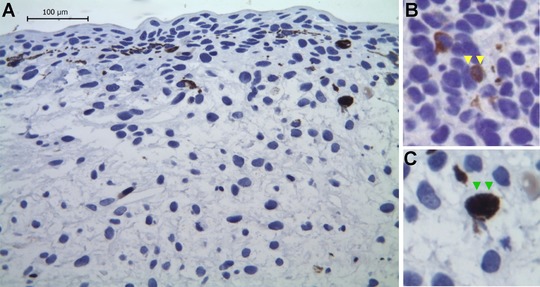
Cell proliferation in pigmented blastema 40 days post‐amputation during limb regeneration in *Bolitoglossa ramosi*. (A) Representative image of a blastema showing BrdU immunostaining as a cell proliferation marker; note the very low level of BrdU incorporation in the regenerate. (B) An example of a BrdU‐positive cell (yellow arrows) from another area of a limb regenerate. (C) An example of a eumelanin producing cell (green arrows), which resembles BrdU‐positive cells due to their high pigmentation

We assessed the cell proliferation in the limb regenerate by incorporation of 5‐bromo‐2′‐deoxyuridine (BrdU) at 40 dpa (Fig. 5); the counting was performed in the mesenchymal area of the blastema (Fig. S3). BrdU‐positive cells were counted in serial sections of the regenerate, and we found that, per counting frame, 1.4% (two to three BrdU‐positive cells) were BrdU positive on average (Fig. S4), which suggests a very low rate of cell proliferation during this stage. In addition, due to high pigmentation, several melanocytes were present throughout the limb blastema (Fig. 5A). These pigmented melanocytes resembled the BrdU‐positive cells but were clearly distinguishable at higher resolution (Fig. 5B, C).

### 2.4 Genome size (*C*‐value) calculation

Four independent samples were performed to determine the genome size. The *C*‐value of control chicken red blood cells was 1222 Mb (1.2 Gb), whereas for *B. ramosi* the value was 25,547 Mb (26 Gb). In the genome size database (Gregory, 2016) to date, the *C*‐value for 20 species belonging to the genus *Bolitoglossa* sp. have been reported, ranging from 42 to 68 Gb; the majority of these values were obtained using the Feulgen densitometry assay. However, in the literature, *C*‐value reports range from 21 to 76 Gb (Mizuno & Macgregor, 1974).

## 3 DISCUSSION

There are approximately 695 species of salamanders that are distributed across 10 families (AmphibiaWeb, 2017). In the field of tissue regeneration, the species that have been used as model organisms for limb regeneration are the axolotl and the newt (Maden, 2008; Voss et al., 2009). Furthermore, the description of limb regeneration in non‐model organisms has also been described (Scadding, 1981). Generally, during limb regeneration the main stages that have been reported are (1) wound healing and dedifferentiation, (2) accumulation and growth of blastema, and (3) differentiation (Iten & Bryant, 1973). In each stage, the cellular organization exhibited in each species is slightly different, and the time reported to reach digital outgrowth is variable between the phylogenetic distribution of urodeles (Table 1) (Scadding, 1981).

**Table 1:** Comparison of limb regeneration stages in different salamanders

Stage/salamander	*Notophthalmus viridescens* (Iten & Bryant, 1973) (dpa)	*Ambystoma mexicanum* (Tank et al., 1976) (dpa)	*Plethodon dorsalis*, *P. cinereus* (Scadding, 1981) (dpa)	*Desmognathus ochrophaeus* (Scadding, 1981) (dpa)	*Eurycea bislineata* (Scadding, 1981) (dpa)	*Bolitoglossa ramosi* (dpa)
Wound healing	1−5	1−5	1−2	1−2	1−2	1−3
Early blastema	17	15	21	14	15	10
Medium blastema	20	20	24	21	−	20
Pigmented blastema	−	−	−	−	−	40
Late blastema	24	24	−	−	−	60
Palette	28	30	27	28	21	70
Digital outgrowth	44	40	50‐71	34	30	95

Few regenerative studies have been conducted in species of the family Plethodontidae, such as *Plethodon dorsalis* and *P. cinereus*, *Desmognathus ochrophaeus, D. fuscus* and *Eurycea bislineata*. The stage described in the present study, “pigmented blastema” (40 dpa) has not been described in other salamander species. Another notable difference between *B. ramosi* and model salamanders (axolotls and newts) is the time taken to reach the digital outgrowth, which is longer in *B. ramosi* (95 dpa).

The forelimbs of *B. ramosi*, which belongs to the Plethodontidae family, have four webbed digits, which is probably an adaptation for their arboreal life. The skin is highly pigmented and highly vascularized, as in other plethodontid salamanders, to favor air gas exchange (Fig. 2A, Video S1) (Vitt & Caldwell, 2009).

In this study, we analyzed the limb regeneration of *B. ramosi*, identified nine macroscopic stages during limb regeneration, and determined that the time taken to reach the four‐digit stage is longer than reported in other species (Table 1). Our observations on limb regeneration in another species of *Bolitoglossa* sp. confirms that the time taken to obtain digital outgrowth in this genus is longer than those reported in aquatic, terrestrial, and biphasic‐lifestyle salamanders.

The finding of a fully pigmented blastema (40 dpa) is noteworthy, as the histological analysis showed considerable pigmentation under the dermal−epidermal junction (Fig. 3C′). The presence of melanophores and eumelanin has been reported in axolotls and newts during the early blastema stage (Scadding, 1981). In *B. ramosi*, the presence of pigmentation was evident macroscopically in the early blastema (7 dpa). However, at 40 dpa the external appearance of the blastema was entirely dark brown due to high pigmentation. To our knowledge, this observation has not been described in other model salamanders or terrestrial salamanders.

These salamanders are terrestrial dwelling animals, and they appear to use the limb blastema as a support during locomotion on land (Videos S2 and S3). Active secretion of eumelanin could possibly confer support to the blastema cells and stabilize the dermal−epidermal junction. Additionally, melanin is considered a protective shield in the skin of the animal from physical or environmental stimuli (Solano, 2014). Furthermore, melanin has been reported to be bactericidal in anurans (Franco‐Belussi & de Oliveira, 2011) showing an immune response. However, in plethodontids, the function of melanocytes as immune cells has not been described.

In addition to high pigmentation, high accumulation of mature collagen fibers under the wound epithelium has also been observed at this stage (Fig. 4) and could be a reflection of the need for a stiff matrix that could give support and protection to the blastema cells during locomotion in a terrestrial environment. In general, collagen fibers are one of the most abundant proteins of the extracellular matrix, which confers cell protection against mechanical forces (Gelse, Pöschl, & Aigner, 2003). We suggest that the presence of this mature collagen fiber underlying the wound epidermis in *B. ramosi* could be a protection mechanism for blastema cells during locomotion. This observation is interesting because collagen or the composition of the extracellular matrix has been shown to regulate cell proliferation, migration, differentiation, and survival (Pickering, 2001). Collagen also acts as a repository for growth factors, changing their bioavailability and function (Nakagawa, Pawelek, & Grinnell, 1989). However, their excessive synthesis is pathological and negative for the regenerative process (Al‐Qattan, Abd‐Al Wahed, Hawary, Alhumidi, & Shier, 2014).

To assess the proliferation rate in blastema cells, we performed a BrdU proliferation assay at the 40 dpa stage and found that the number of cells in S phase is very low (Figs. 5 and S2). The proliferation index during blastema formation in salamanders has been traditionally measured by 3H‐thymidine incorporation (Tassava & Mccullough, 1978; Tassava & Mescher, 1975); these reports suggested that during blastema formation the mitotic index is low, and during growth phase the proliferation increases. We hypothesize that the 40 dpa blastema has not yet entered the proliferation phase and that the proliferation peak of mesenchymal cells could be at about 60 dpa (late blastema/cone stage), where the proliferation rate index is higher (Monaghan et al., 2014). However, further BrdU labeling experiments at other times of regeneration (e.g., 20 dpa, 60 dpa) are needed to understand the dynamics of cell proliferation during blastema formation in *B. ramosi*.

Our observations show that, despite the smaller genome size of *Bolitoglossa* sp., the time taken to complete the regeneration is unusually long in this group compared to other salamanders. The *C*‐value has been described as an important aspect to consider during limb regeneration and has been implicated in other biological processes such as development, metabolism, cellular differentiation, the cell cycle, and growth (Sessions, 2008; Sessions & Larson, 1987). Additionally, some authors have found a significant positive correlation between genome size and cell size and a negative correlation with cell division rate (Gregory, 2002). To explore this phenomenon, we analyzed the genome size of *B. ramosi*. In previous studies, the genome size was quantified by microdensitometry of Feulgen‐stained red blood cells, but the limitation to this approach is the availability of high‐quality densitometers and time (Gregory, 2016; Hare & Johnston, 2011). Here, we used a flow cytometry approach, which allows for precise quantification of the *C*‐value (Hare & Johnston, 2011).

The genome size found for *B. ramosi* was 25 Gb, which is within those reported for the Bolitoglossine clade (21−76 Gb) (Gregory, 2002). If we compare the genome size of *B. ramosi* with the genome size of plethodontids where limb regeneration has been superficially described (Scadding, 1981) (Table 1), such as *Plethodon cinereus* (20−26 Gb), *Desmognathus ochrophaeus* (14−18 Gb), and *Eurycea bislineata* (20−25 Gb) (AmphibiaWeb, 2017), it is noticed that their genome sizes are very similar to that of *B. ramosi*. If we compared the *C*‐value of *B. ramosi* with newts (35 Gb) and axolotls (30 Gb), it is smaller than those species. In 1987, Sessions and Larson (1987) reported that there is a negative correlation between genome size and limb regeneration in plethodontids. Thus, we suggest that genome size itself could not be the reason for the longer time limb regeneration takes for this species.

Other authors have been focused on the relation of developmental stages, body size, and age with regenerative capacities. In axolotl, the body size or age were negatively correlated with limb regeneration capacities, but in the case of metamorphosed axolotls a reduction in regeneration rate and digit malformations were observed (Monaghan et al., 2014). Another interesting possibility to explain the time to undergo full limb regeneration is to see whether direct development versus indirect development processes have some influence in regenerative capacities.

Thus, in this study we evaluated the morphological features during limb regeneration in a non‐model salamander and found that the formation and features of a limb blastema differ from aquatic and other terrestrial salamanders. However, it is important to clarify that conducting research with wild‐caught specimens has limitations due to the availability of specimens to carry out experiments. Nevertheless, our study presents an important description of limb regeneration in a terrestrial salamander and could contribute to the field of regenerative biology by understanding how this process occurs in other salamander families.

## 4 MATERIALS AND METHODS

### 4.1 Animals and husbandry

All animals used in this research were collected under the Ministerio del Medio Ambiente contract on access to genetic resources number 118−2015. All experimental procedures were approved by the Institutional Animal Care and Use Committee of the University of Antioquia.

Wild adult salamanders (7−10 cm snout to tail) of the species *B. ramosi* were collected by the night‐time visual encounter method (Grover, 2006) in the Andes region of Antioquia, Colombia (6°18′5.00″ N, 75°8′26.24″ W). The age of the specimens was not possible to determine, although we used animals in the range of 7−10 cm body length, which were considered to be adult animals. They were kept in the laboratory with minimum environmental conditions (Arenas, Gómez‐Molina, & Delgado, 2015). Briefly, the animals were kept in plastic containers and the humidity was maintained by periodically spraying the cages with charcoal‐filtered tap water. They were maintained at 18−21°C with a 12L:12D photoperiod cycle and fed with fruit flies (*Drosophila melanogaster)* three times a week.

### 4.2 Surgical manipulation

Animals were anesthetized by immersion in 1% tricaine (Sigma‐Aldrich, St. Louis, MO, USA) before surgical procedures (Arenas et al., 2015). The animals were placed in a Petri dish containing 20 mL tricaine for 4 min. Bilateral−proximal amputations of the forelimb were performed at the mid‐humerus level using microscissors (Fine Precision Tools, Foster City, CA, USA). The protruding bone and muscle were trimmed to get a flat wound surface. Following amputation, the wound was rinsed with 1 mL 0.5% sulfamerazine (Sigma) to avoid infection. Animals were rinsed with abundant water to remove traces of tricaine and were returned to the plastic containers for their recovery.

### 4.3 Stages of limb regeneration

Regeneration stages were examined and digitally recorded by weekly observation under a stereomicroscope (Olympus SZX16, Tokyo, Japan) during a 28‐week period. The images were acquired using a digital camera (MotiCAM 5, Kowloon, Hong Kong) coupled to the microscope.

Seven animals with bilateral amputation were used to measure the length of the regenerate; the growth rate per day was calculated as (growth [μm] in week 2 − growth in week 1)/7, (growth [μm] in week 3 − growth in week 2)/7, etc.

The length of the regenerating tissue was measured using the Motic Images Plus (Version 2.0, Kowloon, Hong Kong) software measuring tool function. Briefly, soon after limb amputation, the remnant limb was measured. Regenerating tissue measurements were made proximal to distal, including both the remnant of the normal limb after amputation and the regenerating tissue.

When the elbow was present, the measurement was taken from the tip of the blastema to the elbow and from the elbow to the proximal level of the limb. Finally, when the digits were present, the measurement was taken from the longest digit to the elbow and from the elbow to the proximal level of the limb. The total length of regenerating tissue was obtained by subtracting the initial remnant limb measurement.

### 4.4 Histochemistry

Normal limb (*n* = 6), wound healing (3 dpa) (*n* = 4), early blastema (20 dpa) (*n* = 4), pigmented blastema (40 dpa) (*n* = 4), and palette (70 dpa) (*n* = 4) were used for histological analysis. Normal limbs and regenerated tissues were fixed in 10% formaldehyde at room temperature for 24 h, followed by dehydration in 50%, 70%, and 95% isopropyl alcohol and clearance with xylene. The samples were embedded in paraffin, and serial 4 μm longitudinal sections were cut on a rotary microtome and stained with hematoxylin and eosin. Normal limbs and 40 dpa blastema were also stained with Masson's trichrome stain. The slides were observed under a Leica DMLB microscope (Leica Microsystems Inc.® , Wetzlar, Germany) and photographed with a Leica EC3 camera.

### 4.5 *In vivo* BrdU labeling assay

5‐Bromo‐2′‐deoxyuridine (BrdU) (Sigma, USA) was used as an S‐phase proliferative marker to label the proliferating cells in the limb regenerate at 40 dpa (*n* = 3). BrdU was injected intraperitoneally (0.25 mg/g body weight) at two pulses (24−48 h), as previously reported, to ensure coverage of two cell cycles (Maden, 1976). The regenerating tissues were harvested at 72 h after BrdU injections. The tissues were collected and processed by immunohistochemistry.

Tissues were embedded in paraffin and sectioned longitudinally at 2−3 μm using a rotary microtome. The UltraVision Quanto Detection System (Thermo, Carlsbad, CA, USA) was used to detect the presence of BrdU‐labeled cells following the manufacturer's recommendations. Briefly, the slides were deparaffinized in xylene and rehydrated in a descending ethanol series. Antigen recovery was performed with citrate buffer (pH 6) at 95°C in a water bath for 20 min. Nonspecific background staining due to endogenous peroxidase was blocked, and the slides were incubated in mouse monoclonal antibody against BrdU (1:200) for 1 h (Kumar, Velloso, Imokawa, & Brockes, 2000) and further reacted with Polymer HRP Quanto (Thermo) for 10 min. The DAB Quanto Chromogen and Substrate System (Thermo) was used for color development. The nuclei were counterstained with hematoxylin, and the slides were dehydrated and mounted with Consul Mount.

The slides were visualized under a Leica DMLB microscope, and the acquired images were analyzed by ZEN 2.1 software (Carl Zeiss, Jena, Germany). The BrdU‐labeled cells were counted in the mesenchymal area in five serial sections of each biological replicate. The software ImageJ (NIH, Bethesda, MD, USA) was used to count the total number of mesenchymal blastemal cells in the counting frame, and the BrdU‐positive cells were counted manually because pigmented cells and BrdU‐positive cells could be confused. The cell proliferation data are presented as a proliferative index ([BrdU+/total cells] × 100).

### 4.6 Genome size (*C*‐value) calculation

Red blood cells (5−10 μL) obtained from the amputation plane of the limbs (*n* = 4) were subjected to flow cytometry. Samples were suspended in ethylenediaminetetraacetic acid (EDTA) (pH 7.4, 0.126 mmol /L) and fixed in methanol overnight. Thereafter, the samples were incubated in a solution of RNase (10 mg/mL), Triton X‐100 (0.1% v/v), and EDTA (0.126 mmol/L) and stained with propidium iodide (0.1 mg/mL) for 30 min. The fluorescence intensity was measured in a BD FACSCanto™ II flow cytometer (BD Biosciences, Franklin Lakes, NJ, USA). Chicken red blood cells (DNA QC particles kit, BD Biosciences, Franklin Lakes, NJ, USA) were used as a control. The genome size was calculated by comparison with the reference control (*Gallus gallus)* using the calculation of Hare and Johnston (2011):

$$GS_{unk}=GS_{ref}*\frac{PI_{fluorUnk}}{PI_{fluorRef}}$$

where GS_unk_ is the unknown genome size; GS_ref _is the reference genome size (1222 Mb); PI_fluorUnk_ is the fluorescent intensity of propidium iodide of the unknown sample; and PI_fluorRef_ is the fluorescent intensity of propidium iodide for the reference sample.

## CONFLICT OF INTEREST

The authors have no conflict of interest to declare.

## Supporting information


**Figure S1**. Limb regeneration stages in *Bolitoglossa vallecula*. (A) At 20 dpa the blastema cells accumulate, and at 40 dpa the blastema is highly pigmented. (B) Regenerating animal 12 weeks post‐amputation, when it reaches the late palette stage. (C) During the first 2 weeks the growth of the regenerate is faster, and later growth slows down corresponding to the differentiation and growth of the limb. Approximately 8 weeks after limb amputation, the formation of digits took place. Limb growth rate per day (e.g., [week 2 − week 1]/7 days) was measured in four animals (A, B, C, D).

**Supp. Video 1. *Bolitoglossa ramosi* red blood cells circulating in a normal limb**. This video shows the high vascularization that is present in the normal limb of *B. ramosi*.

**Supp. Video 2. *Bolitoglossa ramosi* walking on blastema during limb regeneration**. This video shows a terrestrial salamander (*B. ramosi*) using its blastema to walk on a hard plastic surface.

**Supp. Video 3. *Bolitoglossa ramosi* walking on palettes during limb regeneration**. This video shows a terrestrial salamander (*B. ramosi*) using its palettes to walk on a hard plastic surface.
