# Supplementary material for: Limb regeneration in a direct‐developing terrestrial salamander, Bolitoglossa ramosi (Caudata: Plethodontidae): Limb regeneration in plethodontid salamanders
Source: Regeneration (Oxf). 2017 Dec 6;4(4):227–35. doi: 10.1002/reg2.93 (PMC5743783; doi:10.1002/reg2.93)
Supplement: Supplementary file 1 — Figure S1. Limb regeneration stages in Bolitoglossa vallecula. (A) At 20 dpa the blastema cells accumulate, and at 40 dpa the blastema is highly pigmented. (B) Regenerating animal 12 weeks post‐amputation, when it reaches the late palette stage. (C) During the first 2 weeks the growth of the regenerate is faster, and later growth slows down corresponding to the differentiation and growth of the limb. Approximately 8 weeks after limb amputation, the formation of digits took place. Limb growth rate per day (e.g., [week 2 − week 1]/7 days) was measured in four animals (A, B, C, D). [file REG2-4-227-s001.docx]

**Supporting Information**


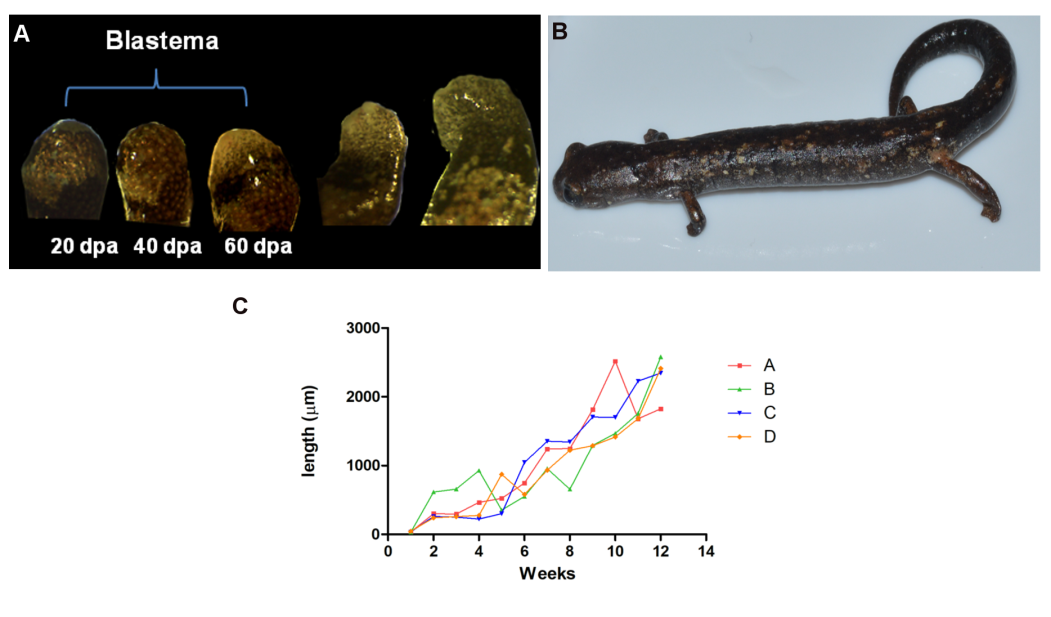


**Supp. 1. Limb regeneration stages in *Bolitoglossa vallecula*. A)** At 20 dpa, the blastema cells accumulate, and at 40 dpa, the blastema is highly pigmented. **B)** Regenerating animal 12 weeks post-amputation, when it reaches the late palette stage. **C)** During the first two weeks, the growth of the regenerate is faster, and later on, the growth diminishes corresponding with the differentiation and growth of the limb. Approximately 8 weeks after limb amputation, the formation of digits took place. Limb growth rate per day (e.g., week 2 - week 1]/7 days), the growth rate was measure in four animals (A, B, C, D).


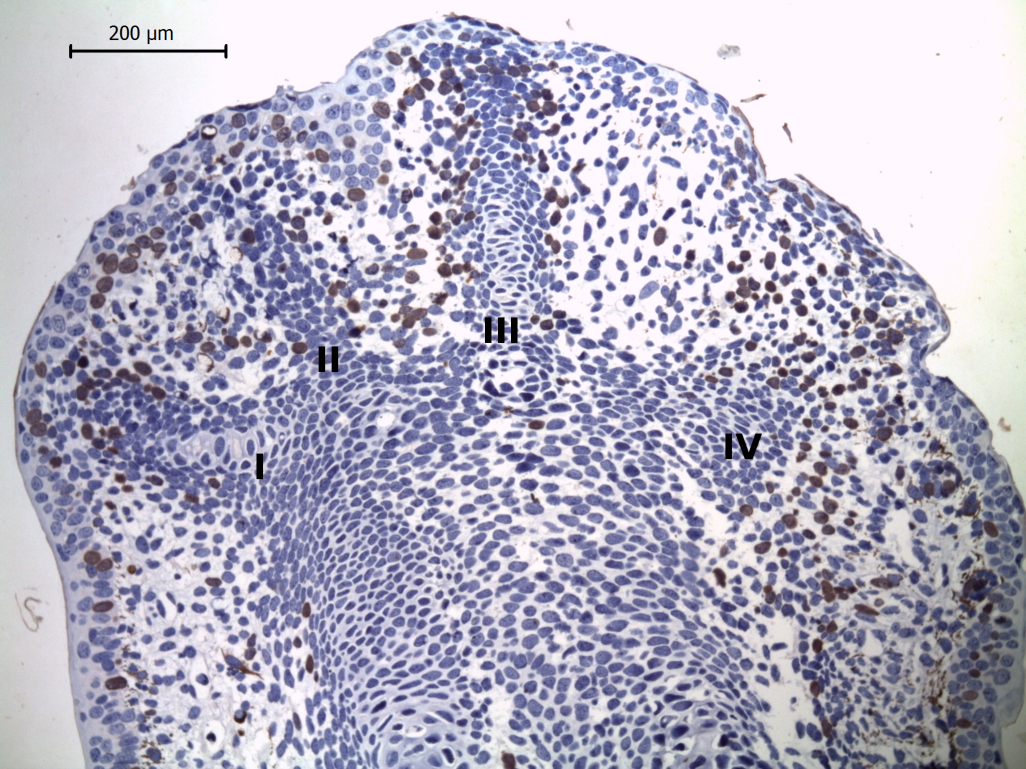


**Supp. 2. Regenerate at the palette stage (70 days post-amputation) in *Bolitoglossa ramosi*.** In the late regenerate, the extension of digit elements I-IV is clearly identifiable, with high condensation of chondrocytes. The cells in the mesenchyme and interdigital zone incorporate BrdU, indicating high cell proliferation in this area. The section is counterstained with hematoxylin. The brown nuclei indicate BrdU-positive cells.


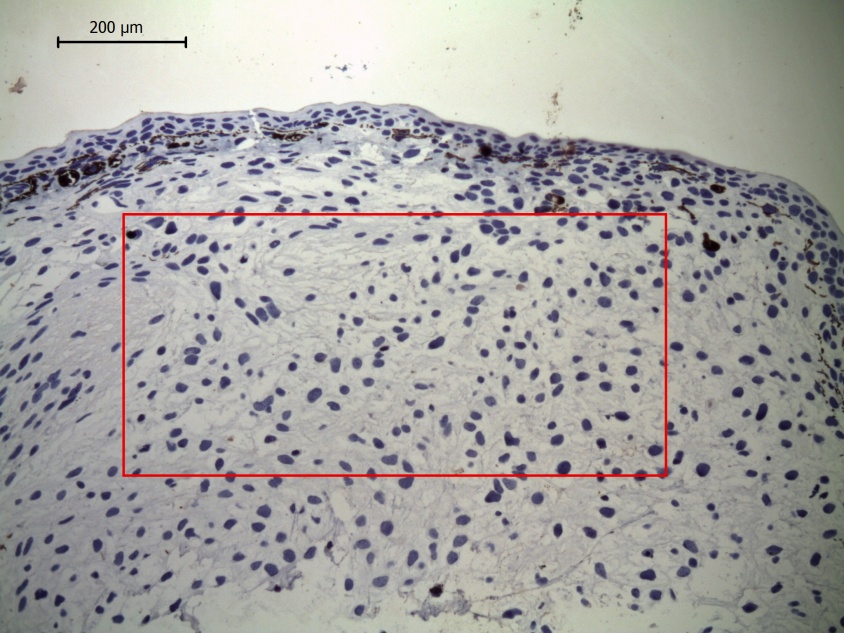


**Supp. 3. Area where the counting of positive BrdU cells was performed.** 10X image of pigmented blastema at 40 days post-amputation during limb regeneration in *Bolitoglossa ramosi*; the red rectangle indicates that the counting of BrdU-positive cells was performed in the mesenchymal blastema

| **Replicate** | **# total cells** | **# total BrdU cells** | **BrdU cells/Total cells (%)** |
| --- | --- | --- | --- |
| **Replicate 1** |  |  |  |
| Section 1 | 194 | 6 | 3.1 |
| Section 2 | 169 | 6 | 3.6 |
| Section 3 | 161 | 4 | 2.5 |
| Section 4 | 188 | 4 | 2.1 |
| **Average** | **178** | **5** | **2.8** |
| **Replicate 2** |  |  |  |
| Section 1 | 232 | 3 | 1.3 |
| Section 2 | 218 | 1 | 0.5 |
| Section 3 | 218 | 0 | 0.0 |
| Section 4 | 235 | 0 | 0.0 |
| **Average** | **225.75** | **1** | **0.4** |
|  |  |  |  |
| **Replicate 3** |  |  |  |
| Section 1 | 117 | 1 | 0.9 |
| Section 2 | 131 | 1 | 0.8 |
| Section 3 | 123 | 1 | 0.8 |
| Section 4 | 147 | 2 | 1.4 |
| **Average** | **129.5** | **1.25** | **1.0** |

**Supp. 4. Table of counts for BrdU-positive cells in the regenerative tissues at 40 dpa in *Bolitoglossa ramosi*.** Three biological replicates were processed for the *in vivo* BrdU labeling assay during the pigmented blastema stage (40 dpa). For each replicate, four sections were counted for the total number of cells in the mesenchymal area and cells positive for BrdU. The percentage of cells positive for BrdU was obtained using the following calculation: (#BrdU cells/Total cells) x 100.

**Supp. Video 1.** ***Bolitoglossa ramosi* red blood cells circulating in a normal limb.** This video shows the high vascularization that is present in the normal limb of *B. ramosi.*

**Supp. Video 2. *Bolitoglossa ramosi* walking on blastema during limb regeneration.** This video shows a terrestrial salamander (*B. ramosi*) using its blastema to walk on a hard plastic surface.

**Supp. Video 3. *Bolitoglossa ramosi* walking on palettes during limb regeneration.**

This video shows a terrestrial salamander (*B. ramosi*) using its palettes to walk on a hard plastic surface.
